# Liquid–Liquid Phase‐Separated Systems from Reversible Gel–Sol Transition of Protein Microgels

**DOI:** 10.1002/adma.202008670

**Published:** 2021-07-08

**Authors:** Yufan Xu, Runzhang Qi, Hongjia Zhu, Bing Li, Yi Shen, Georg Krainer, David Klenerman, Tuomas P. J. Knowles

**Affiliations:** ^1^ Yusuf Hamied Department of Chemistry University of Cambridge Cambridge CB2 1EW UK; ^2^ Cavendish Laboratory University of Cambridge Cambridge CB3 0HE UK; ^3^ School of Chemical and Biomolecular Engineering The University of Sydney Sydney New South Wales 2006 Australia

**Keywords:** all‐aqueous emulsions, biomolecular condensates, elongated microgels, gel–sol transition, gelatin, macromolecular crowding, liquid–liquid phase separation

## Abstract

Liquid–liquid phase‐separated biomolecular systems are increasingly recognized as key components in the intracellular milieu where they provide spatial organization to the cytoplasm and the nucleoplasm. The widespread use of phase‐separated systems by nature has given rise to the inspiration of engineering such functional systems in the laboratory. In particular, reversible gelation of liquid–liquid phase‐separated systems could confer functional advantages to the generation of new soft materials. Such gelation processes of biomolecular condensates have been extensively studied due to their links with disease. However, the inverse process, the gel–sol transition, has been less explored. Here, a thermoresponsive gel–sol transition of an extracellular protein in microgel form is explored, resulting in an all‐aqueous liquid–liquid phase‐separated system with high homogeneity. During this gel–sol transition, elongated gelatin microgels are demonstrated to be converted to a spherical geometry due to interfacial tension becoming the dominant energetic contribution as elasticity diminishes. The phase‐separated system is further explored with respect to the diffusion of small particles for drug‐release scenarios. Together, this all‐aqueous system opens up a route toward size‐tunable and monodisperse synthetic biomolecular condensates and controlled liquid–liquid interfaces, offering possibilities for applications in bioengineering and biomedicine.

## Graphical abstract

Droplet microfluidics is facilitated to engineer liquid proteinaceous materials of controllable size and dispersity at the microscale, through all‐aqueous liquid–liquid phase separation. The protein condensates are highlighted by their morphological evolution, fusion, and the diffusion of nano‐/microspheres in the materials, which would have implications in temperature sensing, drug delivery, and tissue engineering for healthcare.

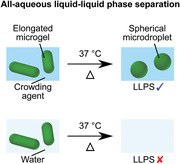

## 1 Introduction

Phase transitions have become general guiding principles for understanding the spatial organization of biological matter in cells and have the potential to aid in the development of soft materials for biomedical studies.^[^ ^1^, ^2^, ^3^, ^4^ ^]^ To date, sol–gel transitions (gelation) have been studied and utilized for the fabrication of materials like soft hydrogels in tissue engineering and also have high relevance for understanding the onset and development of protein‐aggregation diseases.^[^ ^1^, ^2^, ^3^, ^4^, ^5^, ^6^, ^7^, ^8^ ^]^ Recently, there is a growing interest in developing materials formed by liquid–liquid phase separation (LLPS), which is found at the heart of a range of biological processes connected to biological function and malfunction.^[^ ^9^, ^10^, ^11^, ^12^, ^13^ ^]^ The processes underlying the formation of liquid‐state and gel‐state proteins can involve different physicochemical mechanisms, and liquid‐state materials are a key category of functional biological structures as well as an important complement of conventional solid or gel materials. Protein‐rich phases in LLPS systems further have the potential to act as templates for the fabrication and manipulation of biomolecular materials at the microscale. Such liquid states of matter also play key roles in the understanding of the function of structures and processes as diverse as membraneless organelles in living cells, silk spinning by arthropods, as well as the formation of insoluble aggregates associated with neurodegeneration and cancer.^[^ ^11^, ^12^, ^14^, ^15^, ^16^, ^17^ ^]^

LLPS systems can act in many cases as precursors of protein gels or aggregates, but the formation of liquid phases from solid phases (i.e., gel–sol transition) has not been widely studied in the laboratory.^[^ ^12^, ^18^, ^19^ ^]^ Factors such as pH, salt, temperature, ionic strength, and macromolecular crowding agents can impact LLPS.^[^ ^12^, ^17^, ^19^ ^]^ More generally, protein‐rich phases can arise from the condensation from solution; the spontaneous formation of protein‐rich phases typically proceeds by nucleation and growth, and thus a high level of monodispersity of synthetic condensates is challenging to achieve.^[^ ^10^, ^12^ ^]^ We show that this limitation can be circumvented through generating such systems from microgel precursors.

Here, we describe a two‐step bioengineering approach to constructing a highly monodisperse LLPS system by harnessing the thermoresponsive gel–sol transition of gelatin resulting in a demixed all‐aqueous state. First, gelatin microgels were generated on a microfluidic device followed by demulsification into water. Second, these demulsified gelatin microgels underwent a gel–sol transition in aqueous crowding agents at elevated temperature, producing a demixed LLPS system. Monodisperse protein‐rich phases could be achieved through controlling the population density and the size distribution of the precursor microgels. We showed the heat‐triggered shape evolution of the elongated microgels and the fusion of the protein‐rich droplets through time‐lapse optical microscopy highlighting the liquid nature of the protein‐rich phases. As a control, enzymatically crosslinked microgels were thermostable and thus did not turn into liquid. The all‐aqueous LLPS systems developed in this study can shed light on the phase‐separation and phase‐transition behaviors of biocompatible multiphase‐based materials and more on the advancement of 3D printing and drug release for regenerative medicine and temperature sensing.

## 2 Results

**Figure 1:**
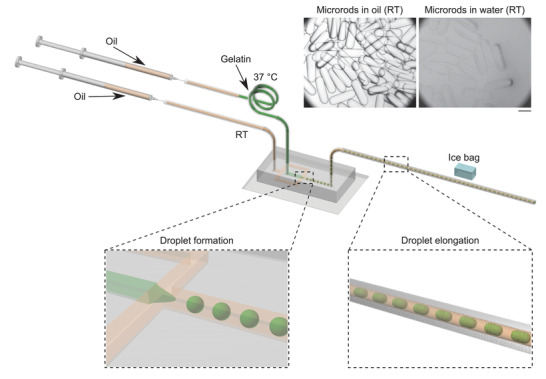
Generation of physically crosslinked protein microrods with a microfluidic approach. An inlet tubing for the gelatin solution was heated to 37 °C; an inlet tubing for the oil phase was placed at room temperature (RT; 25 °C); an outlet tubing was placed under an ice bag for the elongation and physical crosslinking of gelatin microdroplets in oil. The microscopy images at top right show the microrods (elongated microgels) in oil and water at RT. Scale bar: 500 μm.

**Figure 2:**
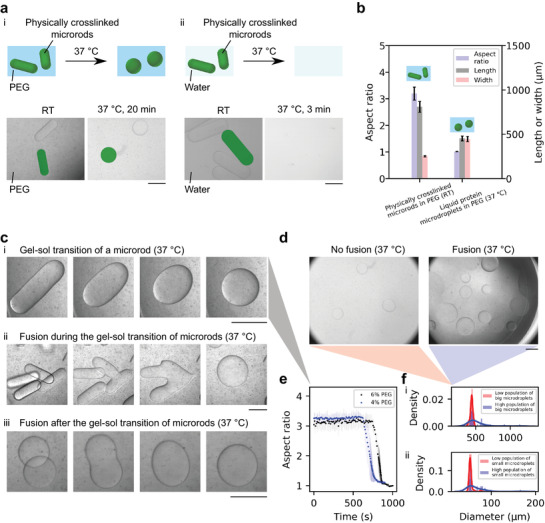
An LLPS system from the gel–sol transition of protein (gelatin solution) in a macromolecular crowding agent (PEG solution). a) Schematics and microscopy images of the formation of the LLPS system from the gel–sol transition of physically crosslinked protein microrods in PEG solution at 37 °C (i). Scale bar: 500 μm. Schematics and microscopy images of a control study on the dissolution of the physically crosslinked microrods in water at 37 °C (ii). Scale bar: 500 μm. b) Geometrical characterisation of physically crosslinked microrods (RT) and liquid protein microdroplets (37 °C), both in PEG solution. Sample size for each: 30. See Figure S1, Supporting Information. c) Gel–sol transition of physically crosslinked microrods in PEG solution. Gel–sol transition of a single microrod (Movie S1, Supporting Information) (i). Fusion during the gel–sol transition of several microrods (Movie S2, Supporting Information) (ii). Fusion after the gel–sol transition of two microrods (Movie S3, Supporting Information) (iii). Scale bar: 500 μm. d) Fusion studies with low (left) and high (right) population of physically crosslinked microrods in PEG solution after incubation at 37 °C. e) Evolution of the aspect ratio of microrods during gel–sol transition (c(i)). f) Monodisperse and polydisperse LLPS from big microgels and small microgels in PEG solution. Big microdroplets are shown in (d) (Figure S2, Supporting Information) (i). Sample sizes: 38 (low microgel population) and 39 (high microgel population). Small microdroplets are shown in Figure S2, Supporting Information (ii). Sample sizes: 40 (low microgel population) and 40 (high microgel population).

Our strategy for forming monodisperse liquid–liquid phase‐separated systems starts with the synthesis of monodisperse microgels through an emulsion templating strategy. To this effect, we used a microfluidic platform with temperature control to maintain the gelatin solution in its liquid state as previously reported (**Figure** **1**).^[^ ^4^, ^20^ ^]^ A microfluidic device with a flow‐focusing junction was used to generate the microrod gels. The gelatin microdroplets were generated at the flow‐focusing junction and were then elongated and physically crosslinked in the thin outlet tubing. Next, these monodisperse microrods in oil were demulsified into water (**Figures** 1 and **2**a(ii); Figure S1a, Supporting Information). There was slight swelling of the microrods during the demulsification because of the water uptake (Figure 1; Figure S1c, Supporting Information).

We set out to develop an all‐aqueous LLPS system from physically crosslinked gelatin microrods by reversing the crosslinking. The microrods were mixed with a crowding agent (poly(ethylene glycol) (PEG)) at room temperature (RT); this mixture was then heated, and the gelatin microrods became liquid microdroplets dispersed in the continuous phase PEG solution at 37 °C, indicating the conversion to an LLPS system (Figure 2a(i)). As a control, the gelatin microrods dissolved in water at 37 °C and formed a well‐mixed solution (Figure 2a(ii)). Geometrical characterization of the elongated microgels and spherical microdroplets was demonstrated (Figure 2b; Figure S1c, Supporting Information). Physically crosslinked microrods stayed elongated in PEG solution at RT and exhibited an aspect ratio of around 3.2, indicating the gel state of the protein phases (Figure 2a(i),b). The liquid microdroplets at elevated temperature, by contrast, exhibited a spherical morphology, corroborating the fact that the gel–sol transition gave rise to PEG–gelatin liquid–liquid interfacial tension (Figure 2a(i),b). The gel–sol transition (Figure 2a(i)) and the dissolution of the microrods (Figure 2a(ii)) were both induced by heating.

Next, a combination of physical and enzymatic crosslinking was used to generate microrods that did not exhibit gel–sol transition at elevated temperature (Figure S1a,b, Supporting Information), as a control study of the physically crosslinked microrods (Figure 2). Previously we have reported different crosslinking regimes, physical or enzymatic, for the templating of protein microgels.^[^ ^4^, ^20^, ^21^ ^]^ Transglutaminase solution was used to enzymatically crosslink the physically crosslinked microrods from their surfaces, and this enzyme catalyses the formation of covalent N ε‐(γ‐glutamyl) lysine amide bonds between the gelatin strands to form a permanent network of polypeptides (Figure S1a,b, Supporting Information).^[^ ^4^, ^20^, ^22^ ^]^ The physically and enzymatically crosslinked microrods did not undergo a gel–sol transition when heated to 37 °C, as they were more thermostable (Figure S1b, Supporting Information) than the physically crosslinked microgels (Figure 2a). The reversible physical crosslinking is key for the thermoresponsive gel–sol transition of gelatin (Figure 2a).

We visualized the thermally induced morphological evolution of physically crosslinked microrods in the environment of macromolecular crowding, demonstrating the gel–sol transition of gelatin. The gel–sol transition of an individual microrod was recorded in a thermostatic chamber (Figure 2c(i); Movie S1, Supporting Information). It took about 10 min to trigger the deformation, and the major shape change took place over ca. 2 min (Figure 2c(i),e). The heat transfer is likely to be the major contributor to this observed lag. Crucially, the protein‐rich compartments could fuse during or after their conversion to the liquid state (Figure 2c(ii),c(iii); Movies S2 and S3, Supporting Information).

The manipulation of the dispersity of the LLPS system can be achieved by avoiding or utilizing the fusion of emulsion droplets. It has been previously reported that bulk mixing of gelatin and PEG solutions typically leads to a polydisperse LLPS emulsion.^[^ ^23^, ^24^ ^]^ This present study, by contrast, proposes a controllable approach to making monodisperse LLPS emulsions by adjusting the population density of gelatin microrods; this approach can be applied to spherical microgels as well (Figure S2, Supporting Information). The monodispersity of the preformed microgels can be transferred to the LLPS droplets, as long as the droplets do not coalesce. Furthermore, the dimension of the condensates can be readily adjusted by the incorporation of microgels with a wide range of sizes (Figure 2f; Figure S2, Supporting Information).

**Figure 3:**
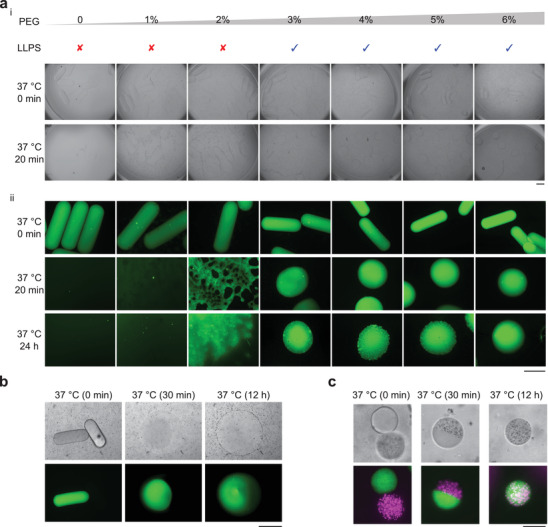
LLPS and nano/microsphere diffusion. a) LLPS with increasing PEG concentration. Bright‐field microscopy (i). Fluorescence microscopy (ii). Scale bar: 500 μm. b) Diffusion of green nanospheres from a nanosphere‐laden droplet to a nanosphere‐free droplet at 37 °C after the fusion of two droplets (see Figure S3iii, Supporting Information). Scale bar: 500 μm. c) Mutual diffusion of green nanospheres and red microspheres between nano/microsphere‐laden droplets at 37 °C after the fusion of two droplets (see Figure S4iii, Supporting Information). Scale bar: 50 μm.

We next focused on the characterization of gelatin microrods in different continuous phases (Figure S1c, Supporting Information). The aspect ratio of the gelatin microrods increased during demulsification, and then remained approximately constant when transferred from water into a PEG solution (Figure S1c, Supporting Information). The microrods swelled when demulsified into water, followed by a further swelling during the enzymatic crosslinking; this implied that crosslinking regimes and temperatures can affect the capabilities of water uptake of the hydrogels (Figure S1c, Supporting Information). The microrods then shrank and dehydrated in PEG solution as a result of osmosis (Figure S1c, Supporting Information); the dehydration was enhanced with ascending PEG concentration (**Figure** **3**a). These results show that the gelatin microrods were responsive to external environmental conditions, a factor that could underlie their use as promising soft and smart building blocks of complex structures for sensing applications.^[^ ^25^, ^26^ ^]^

Next, we set out to characterize the influence of PEG concentration on the LLPS behavior of the gelatin–PEG material system. After a heat‐induced gel–sol transition, spherical gelatin‐rich phases emerged in PEG solutions of high concentrations of PEG (3–6% w/w) (Figure 3a(i)). Green nanospheres were premixed in the gelatin microrods for fluorescence microscopy (Figure 3a(ii)). Wetting of the gelatin‐rich phase was found in 2% PEG (Figure 3a(ii)), yet no clear LLPS was observed in PEG of lower concentrations (0 or 1%) (Figure 3a(i),a(ii)). PEG solutions of higher concentrations have a higher depletion effect to support the LLPS of the protein condensates, in agreement with previous studies.^[^ ^20^, ^23^, ^24^ ^]^

Finally, we demonstrated that the reversible phase transition of the phase‐separated system could be employed to create Janus particles and internal liquid–liquid interfaces within the dispersed condensates. To this end, we generated two populations of microgels, one containing fluorescent nanospheres and the other one devoid of such spheres. Upon fusion of two droplets, initially a liquid Janus structure was formed, followed by slow migration of nanospheres from one protein‐rich droplet to the other protein‐rich droplet (Figure 3b). The adjacent gelatin‐rich microdroplets fused when heated up; the boundary of the droplets was sharp at the beginning, but turned blurred in the end, which indicated the diffusion of nanospheres between droplets (Figure 3b; Figure S3iii, Supporting Information). We also explored the mutual diffusion of nanospheres and microspheres in smaller microdroplets (Figure 3c; Figure S4iii, Supporting Information). A relatively complete diffusion was noticed at 37 °C in droplets after 12 h, while the nano/microspheres barely migrated in gels at RT (Figures S3 and S4, Supporting Information). This finding can be applied as a new path for the production of Janus particles or droplets at varying temperatures (Figure 3b,c; Figures S3 and S4, Supporting Information).

## 3 Discussion

All‐aqueous emulsions are promising models for biomolecular compartmentalization, as these oil‐free emulsions can be used to perform many biological processes that take place in water.^[^ ^13^, ^27^ ^]^ Conventional methods to produce all‐aqueous emulsions include bulk mixing, electrospray, and 3D printing.^[^ ^13^, ^23^, ^27^, ^28^, ^29^, ^30^ ^]^ However, it is challenging to produce monodisperse LLPS emulsions by bulk mixing or 3D printing.^[^ ^23^, ^28^, ^29^ ^]^ The direct on‐chip generation of monodisperse all‐aqueous emulsions is typically challenging as the ultralow all‐aqueous interfacial tension (μN m^−1^) can be much smaller than that of oil–water interfaces (mN m^−1^);^[^ ^9^, ^13^, ^27^, ^31^ ^]^ thus, additional approaches such as mechanical vibrator, hydrostatic pressure, and eletrospray are required to generate a sufficient level of shear force at the all‐aqueous interfaces to avoid jetting.^[^ ^9^, ^13^, ^27^, ^30^, ^32^, ^33^ ^]^ Our study demonstrates a conceptually different route toward monodisperse all‐aqueous LLPS systems through the thermal modulation of the mixture of crowder and preformed monodisperse microgels (Figures 1 and 2).

In this study, we chose gelatin as it is a class of bioactive and biocompatible material, possessing not only a propensity to undergo LLPS in crowded environments but also has tunable physicochemical properties under versatile crosslinking conditions.^[^ ^4^, ^23^, ^28^ ^]^ At higher temperature, gelatin was in liquid state (Figures 1 and 2), which can be explained by the polypeptide chain adopting the conformation of random coils. These random coils transformed into triple helices at RT (Figure 1 and 2), resulting in the formation of physically crosslinked gels. The phase change is reversible as a result of the weak physical crosslinking from intermolecular forces such as hydrogen bonds, van der Waals forces, electrostatic or hydrophobic interactions (Figure 2a).^[^ ^34^, ^35^, ^36^ ^]^ The abovementioned switch of molecular conformations serves as the main mechanism underlying the reversible sol–gel transition of gelatin driven by thermal stimuli, enabling the use of gelatin in a suitable form at various stages of fabricating our all‐aqueous LLPS model.^[^ ^4^, ^20^, ^22^ ^]^ By contrast, PEG solution remained in liquid state throughout the present study. The elongated microgels with reversible phase transition capabilities in this present study complement previous thermostable protein assemblies such as gelatin methacrylate microrods, silk microcylinders, and elongated droplets containing amyloid fibrils.^[^ ^37^, ^38^, ^39^ ^]^ Gelatin and its derivatives have been widely used as the analogues of collagen that is a major component of extracellular matrix proteins.^[^ ^4^, ^22^, ^37^, ^40^, ^41^ ^]^ Gelatin can undergo reversible sol–gel transitions, and it can be processed at physiological temperature and neutral pH at multiple scales;^[^ ^4^, ^20^ ^]^ it inherits cell adhesive motifs as Arg–Gly–Asp from native collagen.^[^ ^4^, ^42^ ^]^ It is of crucial advancement to fabricate all‐aqueous systems with gelatin as collagen substitutes for artificial and miniaturized extracellular matrix platforms for tissue regeneration, pathology research, and drug screening or testing.^[^ ^9^, ^43^, ^44^ ^]^

A recent study has demonstrated that LLPS‐formed droplets can serve as the nucleation precursors that can influence the polymorphism of the co‐assembly resulting in nanosheets, nanoflakes, and nanorods;^[^ ^45^ ^]^ intriguingly, photo‐ or thermally induced phase transitions from these co‐assembled nanosheets (solid) to droplets (liquid) were observed.^[^ ^45^ ^]^ The morphological evolution (Figure 2) of the protein‐rich phase in our present study corresponds to that of the co‐assembly.^[^ ^45^ ^]^ A key focus of our study is the formation of monodisperse protein microgels on chip, followed by the formation of monodisperse LLPS system by modulating the microgel population in the crowding agent; the monodispersity would be unattainable without microfluidic assistance (Figure 2d,f; Figure S2, Supporting Information).

## 4 Conclusion

We have explored a new route for the rapid synthesis of an all‐aqueous LLPS system using phase transition and separation of extracellular protein substitutes in macromolecular crowding. We developed liquid–liquid phase‐separated models with tunable dispersity from thermosensitive microgels in an aqueous crowding agent. The gel–sol transition of these gelatin microrods was demonstrated by their morphological evolution in the crowder, further supported by their fusion behaviors. The morphological evolution of the dispersed phases could open up a new route for the characterisation of gel–sol transition during LLPS. Fusion of the microdroplets could enable the modulation of the dispersity of the LLPS system. Enzymatically crosslinked microgels, as a control, were thermostable and did not support the formation of LLPS systems. The ability to generate highly monodisperse liquid–liquid phase‐separated systems opens up possibilities to use such materials in bioengineering, encapsulation, and delivery applications.

## 5 Experimental Section

### Generation of Physically Crosslinked Microrods

A gelatin solution (w/w, 10%) and a fluorosurfactant‐laden oil phase were prepared as previously reported.^[^ ^4^ ^]^ A hydrophobic microfluidic device (i.e., a droplet maker containing two inlets and one outlet) was made by softlithography technique.^[^ ^18^, ^46^, ^47^ ^]^ An inlet tubing containing the gelatin solution was heated to 37 °C or above, and an inlet tubing containing oil continuous phase was placed at RT;^[^ ^4^ ^]^ an outlet tubing was cooled down by an ice bag. A digital neMESYS pump system (CETONI GmbH, Korbussen, Germany) was used to inject the gelatin solution and the oil phase into the microfluidic device. The microdroplets were formed at the flow‐focusing junction of the microfluidic device at 37 °C, and then elongated and physically crosslinked in the outlet tubing covered by the ice bag. The elongated microgels were demulsified with 10% 1H,1H,2H,2H‐perfluoro‐1‐octanol at RT or or below. 1H,1H,2H,2H‐perfluoro‐1‐octanol was kept in a fumehood. Physically crosslinked microrods were stored in Milli‐Q water.

### Generation of Physically and Enzymatically Crosslinked Microrods

A transglutaminase solution (w/w, 2%) was prepared by dissolving transglutaminase powder (Special Ingredients Ltd., Chesterfield, UK; product of Spain) in Milli‐Q water at RT.^[^ ^4^, ^20^ ^]^ The physically crosslinked microrods were soaked in the transglutaminase solution at RT for 6 h. Then Milli‐Q water was used to exchange the transglutaminase solution. Physically and enzymatically crosslinked microrods were stored in Milli‐Q water.

### Dissolution of the Microrods

The physically crosslinked microrods and the physically and enzymatically crosslinked microrods in Milli‐Q water in a 96‐well plate were heated to 37 °C. Bright‐field images were taken with a high‐speed camera (MotionBLITZ EoSens Mini1‐1 MC1370, Mikrotron, Unterschleissheim, Germany) on a microscope (Oberver.A1, Axio, Zeiss, Oberkochen, Germany).

### Gel–Sol Transition of Microrods in a Crowding Agent

A PEG solution (w/w, 6%) was prepared by dissolving PEG powder (molecular weight 300 000; Sigma‐Aldrich Co Ltd., MO, USA; product of USA) in Milli‐Q water at 50 °C with magnetic stirring for 8 h.^[^ ^20^ ^]^ The physically crosslinked microrods and the physically and enzymatically crosslinked microrods were respectively mixed in the PEG solution at RT, and the final PEG concentration was 5.4% (w/w). The microrod–PEG mixtures were heated to 37 °C in a 96‐well plate; bright‐field images were taken with the abovementioned high‐speed camera and microscope. Videos were taken on a microscope in a homemade thermostatic chamber at 37 °C.

### LLPS Depending on PEG Concentration

PEG solutions with increasing concentrations (0%, 1%, 2%, 3%, 4%, 5%, and 6%) were used to mix with gelatin microrods. The final PEG concentrations in the mixtures were 0%, 0.9%, 1.8%, 2.7%, 3.6%, 4.5%, and 5.4%. The mixtures were heated to 37 °C in 96‐well plates. 1) Bright‐field images were taken with the abovementioned high‐speed camera and microscope. 2) For another experiments, green nanospheres (200 nm, 1% solids, Fluoro‐Max, Thermo Scientific, CA, USA) were premixed in the gelatin (1:20, v/v). Fluorescence images of the microgels were taken with a CCD camera (CoolSNAP MYO, Photometrics, AZ, USA) on a microscope (Oberver.A1, Axio, Zeiss, Oberkochen, Germany); a 49001 filter (excitation wavelength 426–446 nm, emission wavelength 460–500 nm) was used with a compact light source (HXP 120 V, Leistungselektronik Jena GmbH, Jena, Germany).^[^ ^4^ ^]^

### Diffusion of Nano/Microspheres between LLPS‐Formed Droplets

1) Diffusion of nanospheres. Two categories of microrods were prepared by the abovementioned methods, one with the abovementioned green nanospheres (200 nm; v/v, 1:20) and the other without the green nanospheres. The two categories of microrods were mixed at 1:1 ratio and then mixed with 6% PEG solution. The PEG concentration was 5.4% in the final mixture. The mixture was heated to 37 °C in a 96‐well plate. Bright‐field and fluorescence images were taken with the abovementioned imaging facilities. 2) Diffusion of nano/microspheres. For mutual diffusion studies, the abovementioned green nanospheres (200 nm; 1% solids) and red microspheres (1 μm; 1% solids, Fluoro‐Max, Thermo Scientific, CA, USA) were respectively encapsulated in spherical microgels (v/v, 1:20). These spherical microgels were prepared smaller than the microrods at a microfluidic flow‐focusing junction, and no ice bag was used. Fluorescence images of the microgels were taken with the abovementioned CCD camera and microscope; the abovementioned 49001 filter and a 49004 filter (excitation wavelength 532–557 nm, and emission wavelength 570–640 nm) were, respectively, used with the abovementioned compact light source.^[^ ^4^ ^]^

## Conflict of Interest

The authors declare no conflict of interest.

## Author Contributions

Y.X. and R.Q. contributed equally to this project. Y.X. and T.P.J.K. conceived and designed the project. Y.X., R.Q., and H.Z. performed the experiments. B.L. and D.K. assisted in the imaging in Figure 2c(i,ii). Y.X. and R.Q. analyzed the data. Y.S. and G.K. provided advice on the project. Y.X. wrote the manuscript, and all authors commented on the manuscript.

## Supporting information

Supporting Information

Supplemental Movie 1

Supplemental Movie 2

Supplemental Movie 3

## Data Availability

The data that support the findings of this study are available from the corresponding author upon reasonable request.
