# Supplementary material for: Liquid–Liquid Phase‐Separated Systems from Reversible Gel–Sol Transition of Protein Microgels
Source: Adv Mater. 2021 Jul 8;33(33):2008670. doi: 10.1002/adma.202008670 (PMC11468722; doi:10.1002/adma.202008670)
Supplement: Supplementary file 1 — Supporting Information [file ADMA-33-2008670-s003.pdf]

© 2021 Wiley-VCH GmbH

ADVANCED MATERIALS

Supporting Information

for *Adv. Mater.,* DOI: 10.1002/adma.202008670

Liquid–Liquid Phase-Separated Systems from Reversible Gel–Sol Transition of Protein Microgels

*Yufan Xu, Runzhang Qi, Hongjia Zhu, Bing Li, Yi Shen, Georg Krainer, David Klenerman, and Tuomas P. J. Knowles**

Supporting information

Liquid–liquid phase-separated systems from reversible gel–sol transition of protein microgels

Yufan Xu, Runzhang Qi, Hongjia Zhu, Bing Li, Yi Shen, Georg Krainer, David Klenerman, Tuomas P. J. Knowles*

Y. Xu, R. Qi, H. Zhu, B. Li, Dr. Y. Shen, Dr. G. Krainer, Prof. D. Klenerman, Prof. T. P. J. Knowles
Yusuf Hamied Department of Chemistry, University of Cambridge, Cambridge, CB2 1EW, UK.
*e-mail: tpjk2@cam.ac.uk

Prof. T. P. J. Knowles
Cavendish Laboratory, University of Cambridge, Cambridge, CB3 0HE, UK.

Dr. Y. Shen
School of Chemical and Biomolecular Engineering, The University of Sydney, Sydney, NSW 2006, Australia.

LLPS can contribute to intracellular activities that are involved with protein condensates and membraneless organelles, but extracellular matrix (ECM) protein condensates have not been well studied yet (1–4). Phase separation of ECM proteins can be taken for generic mechanisms for the assembly of proteins into fibrillar or polymeric assemblies (5). Understanding the LLPS of ECM proteins, such as elastin and collagen or their substitutes, can contribute to the studies in the aberrant assembly of proteins which can imply pathological consequences and fundamental principles for the optimisation of biomaterials and drugs in the studies of ECM deposition and cancer metastasis (1; 5–9). Intracellular and extracellular coacervates can yield critical insights into cellular behaviours in functional and aberrant biology, and into the abiogenic construction to mimic the inhomogeneity of native environments (4; 10). Intracellular microcompartments, usually represented by membraneless organelles, can have protein-RNA or protein-DNA interactions (3; 11–13); extracellular proteins do not interact with cell nuclei directly. Extracellular proteins are usually sequestrated in hydrated gels or networks, except for liquid or liquid-like situations such as monomers or oligomers in vesicles and newly-secreted or newly-degraded extracellular matrices (14). Adopting ECM proteins or their substitutes as the study objects of LLPS models holds great potential for fundamental physicochemical research and medical applications in drug release and disease models. Discrete and small vehicle/cargoes can potentially have huge drug discovery/delivery implications, such as collagen-gold hybrids, cerium oxide nanoparticles, magnetite nanoparticles, and exosomes (15–21). LLPS models would provide new insights into nanomedicine with therapeutic and pharmaceutical benefits, either within drug-rich phases or at the all-aqueous interfaces. Liquid or liquid-like droplets are piquing curiosity in drug discovery/delivery for neurodegenerative diseases, cancers and more, beyond current knowledge horizons of one-phase or bulk studies (11; 22–24). For example, small-molecule or nanoparticle medicines can be relatively confined locally to allow for controlled drug release to targeted sites at certain rates (10; 18; 24; 25). LLPS systems can inspire the study of the drugs targeting extracellular matrices or fluids, the pharmacodynamics or kinetics with binding assay or diffusion profile, and the effects of drugs on the fusion and growth of organoids in biophysical or biochemical contexts (26–28).

During or after the gel-sol transition, LLPS-formed droplets have great propensity of coalescing or fusion, which could yield bigger droplets or polydispersed LLPS system (29–31). We did not observe the effect of morphology, size or dispersity on the gel-sol transition of gelatin at the micron scale, as such transition was thermally induced in the present study; the approach to controlling the dispersity of the protein-rich phases in the LLPS system is easy to operate with the adjustment of the microgel population.

The purpose of using crowding agent is to introduce depletion force and excluded volume effect and compensate the protein-solvent interaction to make the weak protein intermolecular interaction prominent, which leads to the condensation of protein droplets in

the aqueous continuous phase (4; 25; 29). It should be noted that the gel-sol transition of gelatin is mainly thermo-driven instead of crowder-driven. Previous studies reported the phase separation of gelatin-dextran or gelatin-maltodextrin material systems (32); this indicated that PEG is not the only crowder that triggers LLPS of gelatin. In the present study, the protein gel could firstly dissolve upon the stimulus of elevated temperature, then self-assembled into liquid condensates under the pressure of macromolecular crowding.

We used the morphological evolution of the disperse phases and the diffusion of nano/microspheres in the droplets to support the characterisation of thermo-induced gel-sol transition (Figure 2,3, Main Text). By cooling down the LLPS system from 37 °C to RT, the dispersed phase returned to gel state, while the continuous phase remained liquid (Figure 3b,3c, Main Text; Figure S3,S4); this indicated that there was more gelatin in the dispersed phase (protein-rich) than in the continuous phase (protein-poor). The solubility can be supported by our previous study with a phase diagram demonstrating the one-phase and two-phase zones of gelatin-PEG material system (33). Similarly, solute-rich and solute-poor phases were previously defined in LLPS systems (28; 34; 35). The LLPS model in the present study is promising for the study of gel-sol transition, for example, for the screening of other protein or non-protein materials that may possess such a transition property. Gel-sol transitions of protein or non-protein materials can be induced by external stimuli such as temperature, pH, enzyme, and chelating agents (14; 36–39). A combination of diverse materials and multiple external stimuli can broaden the horizons of all-aqueous LLPS models.

Rational design of immiscible aqueous phases can provide compartmentalised regions for anisotropic and homogeneous cell- or tissue-inspirations, which can progress the understanding of the origin of life in elaborate and sophisticated nature (4; 10; 40). All-aqueous emulsions generated by physical LLPS are usually involved with at least two solute species with one typically enriched in one aqueous phase (4). Dextran-PEG system is perhaps the most prevalent choice for producing all-aqueous emulsions resulting in copious applications (4; 40–42). However, such polysaccharide-crowding material combinations cannot easily elucidate the proteinaceous architecture of beings, unless protein additives were introduced to the all-aqueous systems (4; 40–42). In this present study, we have come up with a simple and facile approach to the formation of all-aqueous protein-crowding LLPS system with the following advancements. 1) The LLPS droplets inherit the monodispersity of microfluidic-generated microgels; the size- and dispersity-adjustable LLPS system is therefore suited for the personalised customisation of microreactors or artificial cell/tissues with soft and dynamic interfaces. 2) Without elaborate laboratory setups, the shape evolution can be readily observed using simple optical microscopes and applied as an indicator for LLPS, simplifying the systematic screening of LLPS conditions and proteins. 3) The LLPS system enables the comparison of the migration of nano/microspheres in gel

or liquid environment at small scales. 4) Our LLPS model features reversible phase transition, which can be used for the exploration of the LLPS and gel states within a single system.

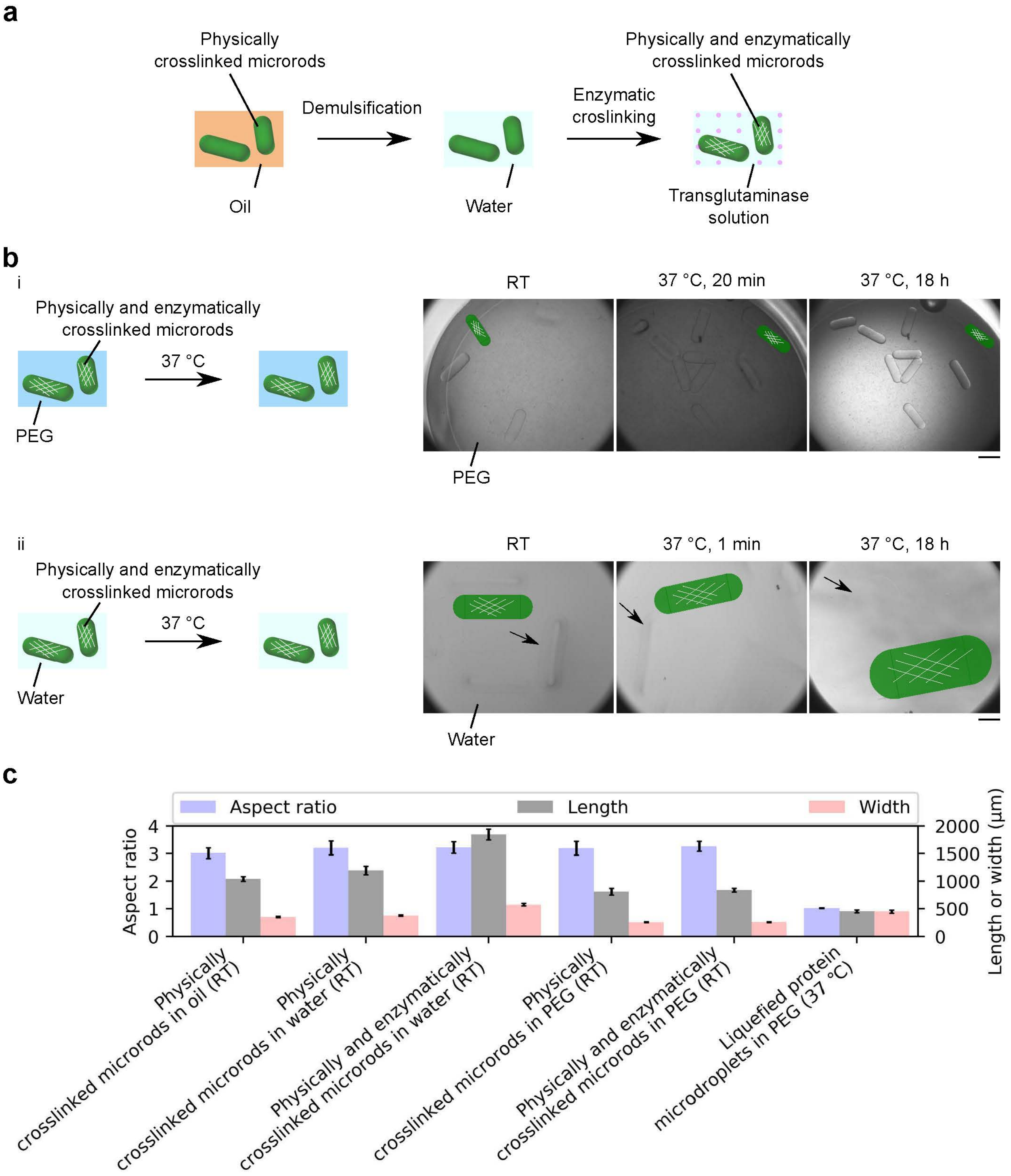

Figure S1 Thermostability of physically and enzymatically crosslinked microrods and geometrical characterisation of protein phases. a) Schematics of the demulsification and enzymatic crosslinking of the protein microrods. b) i,ii, Schematics and microscopy images of physically and enzymatically crosslinked microrods in PEG solution (i) and in water (ii) at 37 °C. Arrows point to gel-water interfaces. Scale bar, 500 µm. c) Geometrical characterisation of protein microrods in oil, water, and PEG solution at RT, and geometrical characterisation of liquefied protein microdroplets in PEG solution at 37 °C. Sample size for each, 30.

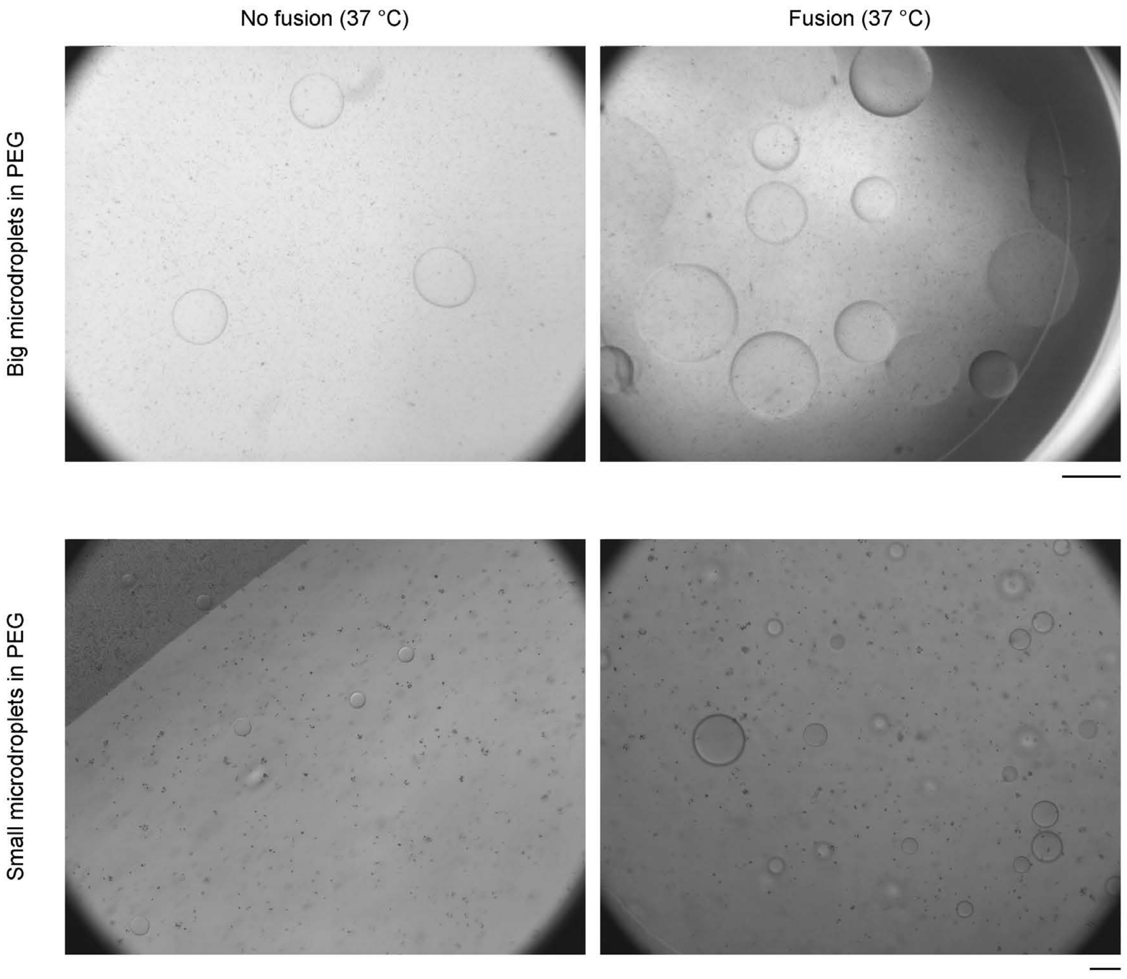

Figure S2 Monodisperse (left images) and polydisperse (right images) of the gelatin droplets in PEG solution at 37 °C. Big microgels (upper images; scale bar, 500 μm) and small microgels (lower images; scale bar, 100 μm) were used.

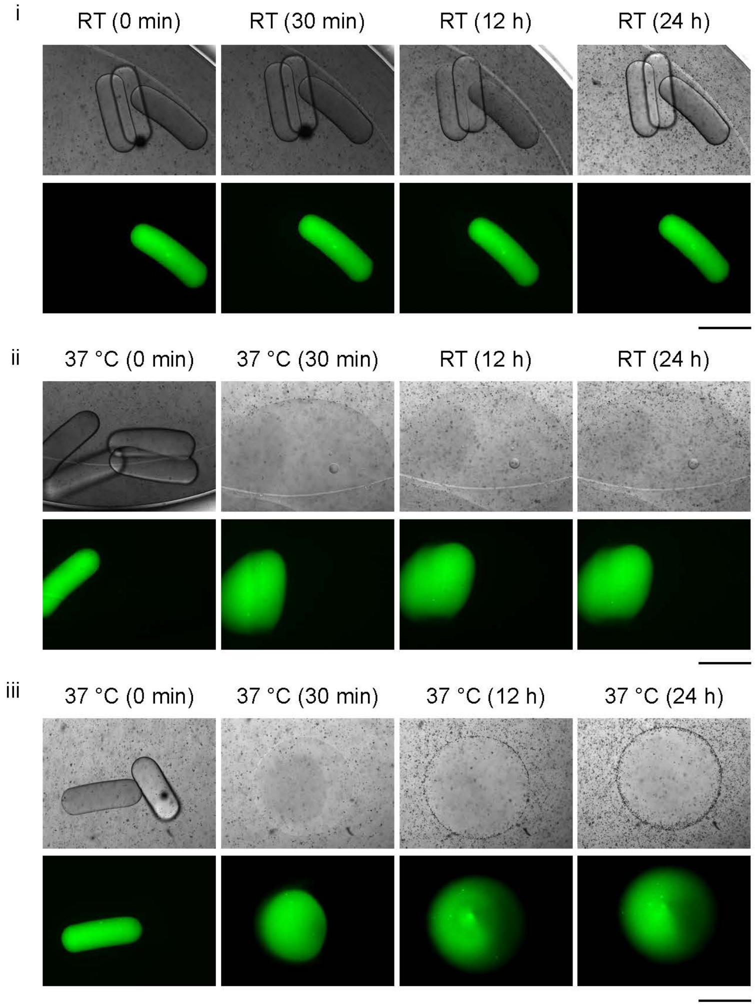

Figure S3 Diffusion of green nanospheres from nanosphere-laden droplets to nanosphere-free droplets. i, The microgel-PEG system was kept at RT for 24 hours. Scale bar, 500 μm. ii, The microgel-PEG system was kept at 37 °C during the first 30 minutes, and was then kept at RT until 24 h. Scale bar, 500 μm. iii, The microgel-PEG system was kept at 37 °C for 24 hours. Scale bar, 500 μm.

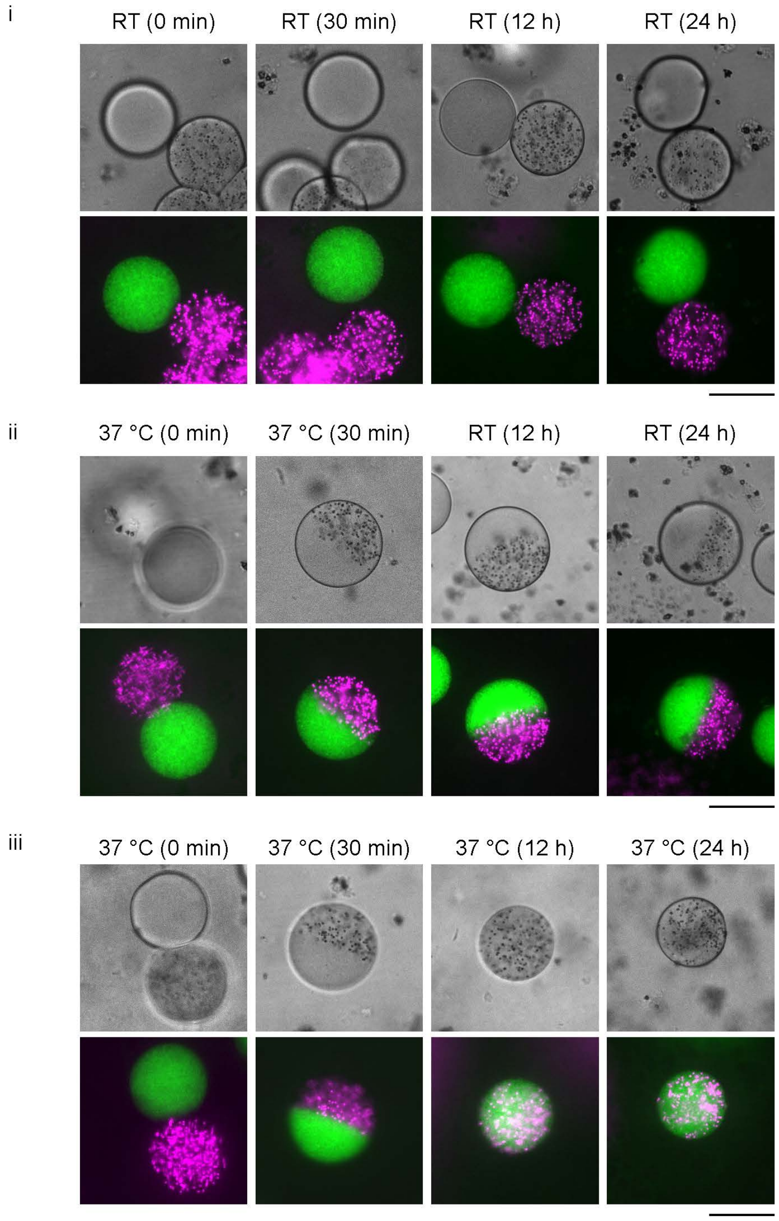

Figure S4 Mutual diffusion of green nanospheres and red microspheres from nano/microsphere-laden droplets. i, The microgel-PEG system was kept at RT for 24 hours. Scale bar, 50 μm. ii, The microgel-PEG system was kept at 37 °C during the first 30 minutes, and was then kept at RT until 24 h. Scale bar, 50 μm. iii, The microgel-PEG system was kept at 37 °C for 24 hours. Scale bar, 50 μm.

References

[1] Y.-P. Chiu, Y.-C. Sun, D.-C. Qiu, Y.-H. Lin, Y.-Q. Chen, J.-C. Kuo, J.-r. Huang. *Nat. Commun.* **2020**, *11*, 1 1.

[2] M. Zeng, Y. Shang, Y. Araki, T. Guo, R. L. Huganir, M. Zhang. *Cell* **2016**, *166*, 5 1163.

[3] M. Feric, N. Vaidya, T. S. Harmon, D. M. Mitrea, L. Zhu, T. M. Richardson, R. W. Kriwacki, R. V. Pappu, C. P. Brangwynne. *Cell* **2016**, *165*, 7 1686.

[4] Q. Ma, Y. Song, W. Sun, J. Cao, H. Yuan, X. Wang, Y. Sun, H. C. Shum. *Adv. Sci.* **2020**, *7*, 7 1903359.

[5] L. D. Muiznieks, S. Sharpe, R. Pomès, F. W. Keeley. *J. Mol. Biol* **2018**, *430*, 23 4741.

[6] N. E. Reticker-Flynn, D. F. B. Malta, M. M. Winslow, J. M. Lamar, M. J. Xu, G. H. Underhill, R. O. Hynes, T. E. Jacks, S. N. Bhatia. *Nat. Commun.* **2012**, *3*, 1 1.

[7] C. Chen, F. Loe, A. Blocki, Y. Peng, M. Raghunath. *Adv. Drug Deliv. Rev.* **2011**, *63*, 4-5 277.

[8] P. Kumar, A. Satyam, X. Fan, E. Collin, Y. Rochev, B. J. Rodriguez, A. Gorelov, S. Dillon, L. Joshi, M. Raghunath, et al. *Sci. Rep.* **2015**, *5*, 1 1.

[9] R. Bascetin, C. Laurent-Issartel, C. Blanc-Fournier, C. Vendrely, S. Kellouche, F. Carreiras, O. Gallet, J. Leroy-Dudal. *Biomaterials* **2021**, *269* 120610.

[10] P. Zhao, B. Yang, X. Xu, N. C.-H. Lai, R. Li, X. Yang, L. Bian. *Adv. Mater.* **2021**, 2007209.

[11] C. P. Brangwynne, C. R. Eckmann, D. S. Courson, A. Rybarska, C. Hoege, J. Gharakhani, F. Jülicher, A. A. Hyman. *Science* **2009**, *324*, 5935 1729.

[12] Y. Shin, C. P. Brangwynne. *Science* **2017**, *357*, 6357.

[13] Y. Shin, Y.-C. Chang, D. S. Lee, J. Berry, D. W. Sanders, P. Ronceray, N. S. Wingreen, M. Haataja, C. P. Brangwynne. *Cell* **2018**, *175*, 6 1481.

[14] A. Jabłońska-Trypuć, M. Matejczyk, S. Rosochacki. *J. Enzyme Inhib. Med.* **2016**, *31*, sup1 177.

[15] S. Li, W. Zhang, H. Xue, R. Xing, X. Yan. *Chem. Sci.* **2020**, *11*, 33 8644.

[16] U. Kauscher, M. N. Holme, M. Björnmalm, M. M. Stevens. *Adv. Drug Deliv. Rev.* **2019**, *138* 259.

[17] H. M. Barriga, M. N. Holme, M. M. Stevens. *Angew. Chem. Int. Ed.* **2019**, *58*, 10 2958.

[18] R. Xing, K. Liu, T. Jiao, N. Zhang, K. Ma, R. Zhang, Q. Zou, G. Ma, X. Yan. *Adv. Mater.* **2016**, *28*, 19 3669.

[19] A. Boey, H. K. Ho. *Small* **2020**, *16*, 21 2000153.

[20] M. Parra-Robert, E. Casals, N. Massana, M. Zeng, M. Perramón, G. Fernández-Varo, M. Morales-Ruiz, V. Puntes, W. Jiménez, G. Casals. *Biomolecules* **2019**, *9*, 9 425.

[21] J. J. Lenders, G. Mirabello, N. A. Sommerdijk. *Chem. Sci.* **2016**, *7*, 9 5624.

[22] A. Mullard. *Nat. Rev. Drug Discov.* **2019**, doi:10.1038/d41573–019–00069–w.

[23] E. Dolgin. *Nat. Biotechnol.* **2021**, *39*, 2 123.

[24] D. Pakravan, G. Orlando, V. Bercier, L. Van Den Bosch. *J. Mol. Cell Biol.* **2020**.

[25] A. A. André, E. Spruijt. *Int. J. Mol. Sci.* **2020**, *21*, 16 5908.

[26] J. A. Brassard, M. Nikolaev, T. Hübscher, M. Hofer, M. P. Lutolf. *Nat. Mater.* **2021**, *20*, 1 22.

[27] Y. Liu, C. Dabrowska, A. Mavousian, B. Strauss, F. Meng, C. Mazzaglia, K. Ouaras, C. Macintosh, E. M. Terentjev, J.-H. Lee, et al. *Adv. Sci.* **2021**, 2003332.

[28] Y. Shen, F. S. Ruggeri, D. Vigolo, A. Kamada, S. Qamar, A. Levin, C. Iserman, S. Alberti, P. St George-Hyslop, T. Knowles. *Nat. Nanotechnol.* **2020**, *15*, 10 841.

[29] C. Yuan, M. Yang, X. Ren, Q. Zou, X. Yan. *Angew. Chem. Int. Ed.* **2020**, *132*, 40 17609.

[30] S. Elbaum-Garfinkle, Y. Kim, K. Szczepaniak, C. C.-H. Chen, C. R. Eckmann, S. Myong, C. P. Brangwynne. *Proc. Natl. Acad. Sci. U.S.A.* **2015**, *112*, 23 7189.

[31] A. Patel, H. O. Lee, L. Jawerth, S. Maharana, M. Jahnel, M. Y. Hein, S. Stoynov, J. Mahamid, S. Saha, T. M. Franzmann, et al. *Cell* **2015**, *162*, 5 1066.

[32] M. F. Butler, M. Heppenstall-Butler. *Food Hydrocoll.* **2003**, *17*, 6 815.

[33] Y. Xu, Y. Shen, T. C. Michaels, K. N. Baumann, D. Vigolo, Q. Peter, Y. Lu, K. L. Saar, D. Vella, H. Zhu, et al. *arXiv preprint arXiv:2009.13413* **2020**.

[34] D. Deviri, S. A. Safran. *bioRxiv* **2021**.

[35] C. Yuan, A. Levin, W. Chen, R. Xing, Q. Zou, T. W. Herling, P. K. Challa, T. P. Knowles, X. Yan. *Angew. Chem.* **2019**, *131*, 50 18284.

[36] C. Bertulli, M. Gerigk, N. Piano, Y. Liu, D. Zhang, T. Müller, T. J. Knowles, Y. Y. S. Huang. *Sci. Rep.* **2018**, *8*, 1 1.

[37] Y. Xu, R. P. Jacquat, Y. Shen, D. Vigolo, D. Morse, S. Zhang, T. P. Knowles. *Small* **2020**, *16*, 32 2000432.

[38] Y. Xu, X. Wang. *Biotechnol. Bioeng.* **2015**, *112*, 8 1683.

[39] S. Rodriguez, H. Lau, N. Corrales, J. Heng, S. Lee, R. Stiner, M. Alexander, J. R. Lakey. *Xenotransplantation* **2020**, *27*, 1 e12554.

[40] Y. Song, T. C. Michaels, Q. Ma, Z. Liu, H. Yuan, S. Takayama, T. P. Knowles, H. C. Shum. *Nat. Commun.* **2018**, *9*, 1 1.

[41] Y. Song, U. Shimanovich, T. C. Michaels, Q. Ma, J. Li, T. P. Knowles, H. C. Shum. *Nat. Commun.* **2016**, *7*, 1 1.

[42] U. Shimanovich, Y. Song, J. Brujic, H. C. Shum, T. P. J. Knowles. *Macromol. Biosci.* **2015**, *15*, 4 501.
